# Neurocognitive functioning in bipolar disorder: What we know and what we don’t

**DOI:** 10.1080/19585969.2022.2042164

**Published:** 2022-06-01

**Authors:** Kamyar Keramatian, Ivan J. Torres, Lakshmi N. Yatham

**Affiliations:** Department of Psychiatry, University of British Columbia, Vancouver, BC, Canada

**Keywords:** Bipolar disorder, neurocognitive functioning, meta-review

## Abstract

**Introduction:** This narrative review of systematic reviews and meta-analyses aims at compiling available evidence in various aspects of neurocognitive functioning in Bipolar Disorder (BD).

**Methods:** We conducted a MEDLINE literature search and identified 38 relevant systematic reviews and metaanalyses.

**Results:** Current evidence suggests that BD is associated with cognitive impairment across multiple domains and during all clinical states. However, there is a considerable cognitive heterogeneity within BD, which cannot be explained by clinical subtypes, and the pattern of neurocognitive impairment in BD overlaps with other psychiatric conditions such as major depression and schizophrenia. Residual depressive symptoms, poor clinical course and higher number of manic episodes may negatively impact cognitive performance, which is a major predictor of general functioning in BD. Evidence from available prospective studies does not support the notion of progressive cognitive decline in BD while some evidence exists to suggest patients may show some improvements in cognitive functioning following the first manic episode. Furthermore, a subset of patients may show premorbid cognitive abnormalities that could signal an early neurodevelopmental aetiology. Preliminary findings from small studies identify potential pro-cognitive effects of Cognitive Remediation, erythropoietin, intranasal insulin, lurasidone, mifepristone, repetitive Transcranial Magnetic Stimulation and transcranial Direct Current Stimulation in BD.

**Discussion:** Longitudinal studies in high-risk individuals can provide a better understanding of the development and progression of neurocognitive impairment in BD. Largescale randomised control trials are needed to compare the pro-cognitive efficacy of various pharmacological and non-pharmacological interventions in different cognitive subgroups of patients at different stages of BD.

## Introduction

Bipolar Disorder (BD) is a psychiatric condition characterised by fluctuating mood episodes called (hypo)mania and depression, interspaced with periods of euthymia. Many patients with BD experience residual symptoms between acute episodes and continue to demonstrate substantial functional and cognitive impairments even during periods of symptomatic remission (Malhi et al. 2007). The past two decades has seen a growing interest in the study of neurocognitive functioning in BD resulting in a rapidly expanding body of literature in this area of research. The exponential growth in this area makes it challenging for clinicians to remain informed on this diverse literature. Therefore, the purpose of this review is to provide clinicians with an overview of neurocognitive functioning in BD by compiling available evidence from systematic reviews and meta-analyses. We begin with reviewing the evidence for neurocognitive impairment across different clinical states as well as the role of demographic and clinical factors that can influence cognitive performance in BD. Next, we discuss the heterogeneity of cognitive functioning in BD, particularly in relation with clinical subtypes of BD and how the pattern and magnitude of neurocognitive impairments in BD compare with schizophrenia and major depression. We then evaluate the development and progression of cognitive impairment in BD. We also examine the role of cognitive impairment in day-to-day and occupational functioning of patients affected by BD, and provide an overview of pharmacological and nonpharmacological interventions that have been studied to improve cognitive performance in patients with BD. We conclude the review by discussing future directions for research.

## Methods

We conducted a MEDLINE literature search using search terms including ‘bipolar disorder’, ‘systematic review’, ‘meta-analysis’, ‘cognit*’, ‘neurocog*’ and ‘neuropsycholog*’ from inception until 21 October 2021. Out of 327 retrieved articles, 38 relevant systematic reviews and meta-analyses were included in this review.

## Results

### What is the pattern and magnitude of neurocognitive impairment during different clinical states?

In a meta-analysis of 26 studies, Robinson et al. (2006) aimed to identify the pattern and magnitude of neurocognitive impairment in patients with BD during remission. The analysis revealed large effect sizes (d ≥ 0.8) for aspects of executive functioning such as category fluency and mental manipulation as well as verbal learning in euthymic patients with BD. Less pronounced deficits were found for immediate and delayed verbal memory, abstraction and set-shifting, sustained attention, response inhibition and psychomotor speed (0.5 ≤ d < 0.8) as well as for verbal letter fluency, immediate memory and sustained attention (0.2 ≤ d < 0.5). The authors also noted significant variation in the criteria used by individual studies to define euthymia and identified residual mood symptoms and psychotropic medications as major confounding factors. Another meta-analysis (Torres et al. 2007) examined 15 most studied cognitive measures in euthymic BD across 39 studies and found medium to large effect size differences across the general domains of attention and processing speed, episodic memory and executive functioning. Similar to the meta-analysis by Robinson et al., they identified the impacts of subclinical mood symptoms and medications as potential confounding factors.

Another meta-analysis (Kurtz and Gerraty 2009) investigated the effects of clinical states on cognitive impairment by analysing 42 studies of patients in euthymia, 13 studies of patients in a manic or mixed state and 5 studies of patients in a depressed state of BD. Consistent with results from earlier meta-analyses, euthymic patients with BD demonstrated neurocognitive impairment across all domains, with effect sizes being in the moderate to large range for most measures. As for the impact of different mood states, patients tested during a manic/mixed or depressed episode exhibited exaggerated cognitive impairment in verbal learning compared to patients tested while in remission. Patients with bipolar depression also showed an increased magnitude of phonemic fluency deficits relative to euthymic patients.

In sum, evidence from multiple studies has consistently shown neurocognitive impairments with moderate to large effect sizes in BD during all clinical states including euthymia. Impairments in verbal memory and aspects of executive functioning appear to be more severe than other cognitive domains.

### What demographic and clinical factors may influence cognitive performance in BD?

A meta-analysis (Mann-Wrobel et al. 2011) investigated the potential moderating effects of demographic and clinical variables on cognitive performance of euthymic patients with BD. Sex and diagnostic rigour of euthymia did not affect cognitive performance; however, there was a reduction in neurocognitive impairment with increased education levels, as well as older age and longer duration of illness. Moreover, among seven neurocognitive tests for which sufficient data were available, i.e., Category Fluency, Letter Fluency, List Learning, Trail Making Test Part A (TMT-A) and Part B (TMT-B), Wisconsin Card Sorting Task (WCST) categories, and WCST perseverative errors, poor clinical course was associated with worse performance on all but two measures, namely, category fluency and letter fluency. A more recently published systematic review (Dauvermann and Donohoe 2019) examined the impact of childhood trauma in neurocognitive functioning of patients with BD. Four individual studies in that review reported an association between greater childhood trauma and poorer performance in general cognitive ability, memory and executive functioning. Association between severity of childhood trauma and cognitive performance has also been reported in healthy participants (HP) and less consistently in patients with schizophrenia (Dauvermann and Donohoe 2019).

In an attempt to adjust for confounding factors and provide a more accurate estimate for effect sizes of cognitive impairment in BD, Bourne et al. performed an Individual Patient Data Meta-Analysis by reanalysing 31 primary studies of euthymic patients with BD and HC as a single sample (Bourne et al. 2013). The results suggested cognitive impairment across all 11 neuropsychological measures after controlling for age, IQ and sex; however, the effect sizes were lower (0.26–0.63) than the ones reported in previous meta-analyses. They also found that residual depressive symptoms and number of manic episodes had small effects on Verbal Learning Test (VLT) and TMT-A. As for medication effects, antipsychotics had negative impact on VLT, while other medications (lithium, antidepressants and anticonvulsants) did not show any effect on cognitive performance.

The potential effect of medications on cognition also deserves more discussion. Current literature on the impact of second-generation antipsychotic medications on cognitive functioning in patients with BD remains inconclusive, as demonstrated by a recently published narrative review (Xu et al. 2020). This might be in part due to the possible differential effects of different antipsychotic medications on cognitive performance (Torrent et al. 2011; Yatham et al. 2017). A meta-analysis by Wingo et al. (2009) suggested that in patients with mood disorders, exposure to lithium was associated with a minor negative impact on verbal learning, memory and creativity and a moderate negative effect on psychomotor performance. The discrepancy in the literature regarding the impact of lithium on neurocognitive functioning in BD might be due to the heterogeneity within BD which, in turn, can lead to varying degrees of response to treatment and impact on cognition. In fact, observational studies have shown that about one-third of patients treated with lithium monotherapy achieve long-term remission (Garnham et al. 2007). These ‘excellent lithium responders’ demonstrate normal neurocognitive functioning compared to patients who respond sub-optimally to lithium (Rybakowski and Suwalska 2010). Lastly, compared to other mood stabilising medications such as lithium, valproate has been shown to be associated with worse cognitive performance (Xu et al. 2020).

Taken together, impairment in cognitive performance in patients with BD cannot be fully accounted for by potential demographic and clinical confounders. History of childhood maltreatment seems to have deleterious effects on cognitive performance in patients with BD, as in HP. Residual depressive symptoms, poor clinical course and higher number of previous manic episodes may have a minor negative effect on performance, while the impact of medications, especially lithium, on cognition varies and may depend on the patient’s clinical response.

### What is the prevalence of neurocognitive impairment in BD?

A systematic review (Cullen et al. 2016) aimed to estimate the prevalence of impairment across various cognitive domains in euthymic adult patients with BD. Their findings suggested wide heterogeneity across studies and domains: Using 1.64 standard deviation (approximately fifth percentile) impairment threshold, prevalence ranges were 5.3%–57.7% for executive functioning, 9.6%–51.9% for attention and working memory, 23.3%–44.2% for speed/reaction time, 8.2%–42.1% for verbal memory and 11.5%–32.9% for visual memory. More recently, Green et al. (2020) systematically reviewed studies that used data-driven clustering methods to determine cognitive subtypes among adult patients with BD. Most of the eight studies that aimed to identify cognitive subgroups in BD revealed a 3-cluster solution with between 14% and 50% of patients with BD being categorised as either high performers or cognitively intact, and other subgroups showing varying degrees of cognitive impairment (selective or moderate vs. global or severe impairment).

Lastly, another recently published systematic review and meta-analysis (Léda-Rêgo et al. 2020) explored the prevalence of functional impairment as measured in patients with euthymic BD with the Functioning Assessment Short Test, which is a validated interviewer-administered instrument that is commonly used in BD studies to assess functional impairment in multiple areas including cognition (Rosa et al. 2007). Meta-analysis of 13 included studies suggested that 49.2% of patients with BD reported subjective difficulties with concentration and memory during euthymia.

Overall, there is a substantial discrepancy across published studies in relation to the estimated prevalence of cognitive impairment in BD. Nonetheless, all studies have identified significant cognitive heterogeneity in BD, i.e., while a sizable subgroup of patients with BD exhibit normal or even higher cognitive performance, most patients exhibit various degrees of impairment. Subjective cognitive complaints are reported by nearly half of patients with euthymic BD.

### Do different clinical subtypes of BD show distinct neurocognitive features?

Can cognitive heterogeneity in BD be explained by clinical heterogeneity? A meta-analysis by Bora aimed to answer this question by investigating the potential effects of history of psychotic features and full manic episodes, on neurocognitive functioning (Bora 2018). The findings indicated that both history of psychotic features and history of full manic episodes, i.e., Bipolar I Disorder (BD-I) were linked to modestly more severe (d = 0.19 and 0.17, respectively) cognitive deficits. Regarding individual cognitive domains, patients with BD-I underperformed relative to those with Bipolar II Disorder (BD-II) in verbal memory, processing speed and executive functioning (d = 0.15–0.26). History of psychosis was associated with more pronounced neurocognition impairment in verbal memory, processing speed, executive functioning, working memory and social cognition (d = 0.12–0.28). Overall, the small effect sizes suggest that most of the cognitive heterogeneity in BD cannot be attributed to the clinical subtypes. Another meta-analysis (Dickinson et al. 2017) aimed to assess executive functioning deficits in BD-I and BD-II. Thirty-six studies were included, and six subdomains of executive functioning, i.e., set shifting, inhibition, planning, verbal fluency, working memory and attention were identified. Overall, the authors were unable to identify consistent findings for differences in executive functioning between BD-I and BD-II patients. For BD-I, impairment was reported in all six subdomains of executive functioning, and BD-II studies reported impairment in verbal fluency, working memory, set shifting and attention compared to HP. As for comparing performance in executive functioning in BD-I versus BD-II, the results were mixed, and no consistent difference between the two groups was identified. Similarly, most studies included in another recently published systematic review (King et al. 2019) reported similar cognitive profile between BD-I and BD-II.

In sum, although comparative cognitive studies are sparce, available evidence from the extant literature suggests that clinical subtypes of BD may show comparable neurocognitive profiles and clinical heterogeneity does not seem to largely account for cognitive heterogeneity in BD.

### Is the pattern of neurocognitive impairment in BD diagnosis-specific?

A number of systematic reviews and meta-analyses have compared neurocognitive functioning between BD and schizophrenia (Bortolato et al. 2015). One of the most recent meta-analyses compared cognitive abilities in first episode BD and first episode schizophrenia (Bora and Pantelis 2015). Both groups showed significant impairments across different cognitive domains and cognitive functioning in first episode BD fell between first episode schizophrenia and HP. Patients with first episode BD significantly outperformed those with first episode schizophrenia in several domains of neurocognitive functioning including processing speed (*d* = 0.33), verbal fluency (*d* = 0.50), verbal memory (*d* = 0.47) and working memory (*d* = 0.35). In addition, current (*d* = 0.63) and premorbid (*d* = 0.50) IQ were found to be significantly higher in patients with first episode BD compared to patients with first episode schizophrenia. However, no significant difference was observed between the two groups in areas such as reasoning, sustained attention and visual memory. These findings were comparable to meta-analyses that included studies of chronic samples with BD and schizophrenia (Krabbendam et al. 2005; Daban et al. 2006; Bora et al. 2009a; Vöhringer et al. 2013; Lynham et al. 2022). Trotta et al. (2015) conducted a systematic review and meta-analysis to investigate premorbid and post-onset cognitive functioning of patients with BD and schizophrenia. The authors concluded that while schizophrenia was associated with significant impairment in premorbid intellectual functioning, BD was associated with small premorbid intellectual functioning deficits only when intellectual assessments were performed retrospectively and not prospectively. Moreover, the magnitude of post-onset cognitive decline was greater in patients with schizophrenia (Trotta et al. 2015).

Another published meta-analysis (Samamé et al. 2017) reviewed 23 reports to compare neurocognitive performance between BD and major depressive disorder (MDD), both during depressive states and euthymia. No difference was observed between the two conditions during depressive states. During euthymia, significantly worse performance (Hedges’g = 0.64) was found in BD compared with major depression only for verbal memory, as measured by list learning tests. No significant between-group differences were detected for other cognitive variables including TMT-A, TMT-B, processing speed, digit symbol coding, response inhibition and cognitive flexibility. Similar findings were reported in a systematic review comparing neuropsychological profiles of MDD and BD during euthymia (Szmulewicz et al. 2017).

Taken together, current evidence suggests that, on average, patients with schizophrenia tend to show more severe impairment than patients with BD, especially in general intelligence, verbal fluency and verbal memory. This may in part be due to the cognitive heterogeneity within each diagnosis and the observation that compared to schizophrenia, more patients with BD belong to the cognitively intact subgroup (Lewandowski et al. 2014; Bora 2016). Also, available data suggests that BD may present with more severe selective deficits in verbal memory relative to MDD.

### When do the neurocognitive deficits in BD first develop?

A systematic review by Daglas et al. (2015) suggested that some degree of cognitive deficit exists after remission from first episode of mania and the most consistently reported finding was impairments in working memory. A meta-analysis by Lee et al. (2014) indicated that after controlling for potential demographic and clinical confounders, patients with first episode BD demonstrated significant deficits in psychomotor speed, attention and working memory and cognitive flexibility (effect size ≥ 0.5) as well as in verbal learning and memory, attentional switching and verbal fluency (effect size 0.20–0.49). Similarly, another meta-analysis by Bora and Pantelis (2015) indicated that patients with first episode BD showed significant impairment in all cognitive domains (*d* = 0.26–0.80) and individual tasks (*d* = 0.22–0.66). Finally, Elias et al. (2017) evaluated cognitive impairment in early onset BD by conducting a systematic review and meta-analysis in euthymic youths with BD-I and BD-II. Findings suggested that euthymic youths with BD showed significant impairments in several cognitive domains including verbal learning, verbal memory, working memory, visual learning and visual memory, with moderate to large effect sizes (Hedge’s g = 0.76 − 0.99). These findings suggest that significant cognitive impairment is already established in adults and youth who have recently recovered from their first BD episode.

Martino et al. (2015) aimed to explore the onset of neurocognitive impairments in BD by performing a systematic review on neurocognitive functioning in the premorbid stage by looking at conscript, birth cohort and high-risk studies, as well as in the first episode of BD. Most studies that assessed neurocognitive functioning in the premorbid stage of BD only reported general intelligence, which was shown to be comparable to that of HP, while one study suggested high premorbid IQ was associated with increased risk for developing mania (Koenen et al. 2009). Only two high-risk studies with small sample sizes reported on specific cognitive domains, with one study suggesting impairments in executive functioning (Meyer et al. 2004) and the other one in visuospatial reasoning and global intelligence (Ratheesh et al. 2013) prior to the onset of BD. Finally, in order to investigate the possibility of cognitive endophenotypes in BD, a meta-analysis was conducted to compare cognitive performances of euthymic patients with BD (45 studies) or unaffected first-degree relatives of patients with BD (17 studies) with HP (Bora et al. 2009b). Unaffected first-degree relatives of patients with BD were found to have similar but less pronounced (small to medium effect sizes) impairments than patients affected by BD in domains such as response inhibition, set shifting, executive functioning, verbal memory and sustained attention. However, unlike patients with BD, unaffected relatives did not show significant impairment in processing speed, visual memory and verbal fluency. More recently, a systematic review of 23 studies of patients with BD and 28 studies of unaffected relatives suggested that patients with BD and, to a lesser degree, unaffected relatives show impairments in attention, processing speed, verbal memory, and verbal fluency (Cardenas et al. 2016). Lastly, a recent meta-analysis (Bora 2017) showed that first-degree relatives of individuals with BD underperform HP in domains such as processing speed, verbal fluency, as well as executive functioning (d = 0.33–0.41).

To summarise, it appears that some degree of cognitive impairment is evident in unaffected first-degree relatives of patients with BD and in the premorbid stage at least in a subgroup of patients with BD. However, the most significant decline in cognitive functioning may occur around the time of the first BD episode.

### Is neurocognitive deficit in BD progressive?

A recently published systematic review and meta-analysis (Szmulewicz et al. 2020) examined the longitudinal course of cognitive performances in patients with recent-onset and late-life BD. Eight longitudinal studies of recent-onset and four studies of late-life BD were included. No significant differences in the longitudinal change in global cognition or specific neurocognitive domains were observed between patients with recent-onset BD and HP (mean follow-up: 17 months). Meta-regression analyses suggested that factors such as length of follow-up, change in manic and depressive symptoms and medication use at baseline had no significant impact on neurocognitive changes over time. Similarly, no evidence for a significant change in cognitive performance was observed during follow-up in patients with late-life BD (mean follow-up: 33 months). These findings were in line with an earlier meta-analysis, which showed the trajectory of neurocognitive functioning in BD was not significantly different from that of patients with schizophrenia and HP over the short-term (mean duration = 1.5 years) or the long-term (mean duration = 5.5 years) follow-ups (Bora and Özerdem 2017). The authors also found evidence for significant improvement in memory and executive functioning of patients with BD at short-term but not long-term follow up. Similar findings have been reported in an earlier systematic review (Cardoso et al. 2015) and meta-analysis (Samamé et al. 2014).

In summary, little evidence exists to suggest a progressive decline in neurocognitive functioning in patients with BD beyond the first episode.

### Is cognitive performance associated with functional outcomes in BD?

A meta-analysis of 22 studies was conducted to evaluate the contribution of cognitive performance to day-to-day functioning of patients with BD (Depp et al. 2012). The results indicated that all cognitive domains are significantly associated with day-to-day functioning ranging from the lowest correlation for Visual Learning and Memory (r = 0.21) to the highest correlation for Working Memory (r = 0.29). The composite cognitive functioning and day-to-day functioning were also significantly correlated (r = 0.33) and the magnitude of the impact of cognitive performance on day-to-day functioning in patients with BD was similar to the one reported in studies of schizophrenia (Fett et al. 2011). Another meta-analysis examined predictors of favourable employment outcomes in patients with BD (Tse et al. 2014). Pooled findings from 14 cross-sectional and 8 longitudinal studies suggested that verbal memory and executive functioning were moderately related to positive employment outcomes. Importantly, cognitive variables as a whole were shown to contribute greater than sociodemographic or clinical variables to employment outcomes. Finally, a systematic of review of 18 cross-sectional and longitudinal studies reported that despite some inconsistencies in the literature, current evidence suggests that cognitive functioning is associated with functional outcomes in both symptomatic and euthymic patients with BD (Baune and Malhi 2015).

Overall, cognitive impairment is one of the main predictors of functional impairment in patients with BD.

### What interventions can improve neurocognitive deficits in BD?

A recently published systematic review of randomised controlled trials (RCTs) aimed to review available evidence relating to pharmacological, neurostimulation, and psychological strategies for the treatment of cognitive impairment in BD (Tamura et al. 2021). Most of the 22 included studies were exploratory in nature, used small sample sizes and had high rates of participant dropout. Six RCTs examined the effect of Cognitive Remediation Therapy in BD (N = 345). Of these, two studies (Demant et al. 2015; Ott et al. 2021) reported negative results for their primary outcomes. Another study (Gomes et al. 2019) developed an intervention called cognitive behavioural rehabilitation by combining elements of cognitive behavioural therapy and Cognitive Remediation and compared it with treatment as usual with time until the new episode as their primary outcome. They reported significantly improved reaction time, visual memory and emotion recognition in cognitive behavioural rehabilitation compared to treatment as usual. Another RCT (Bernabei et al. 2020) suggested that cognitive remediation was superior to the control intervention in improving domains related to executive functioning, attention, memory, and impulse control. Similarly, a more recently published single-blind RCT (Strawbridge et al. 2021) reported significant improvement in working memory and executive functioning as well as in psychosocial functioning and goal attainment in patients with BD who received cognitive remediation compared to treatment as usual. The most robust evidence for the efficacy of cognitive remediation so far comes from the study by Lewandoski et al. who randomised 75 patients with BD with psychosis to a 70-h computerised cognitive remediation program and a dose-matched computer control. Compared to the control intervention, patients who received cognitive remediation showed significant improvements in cognitive performance on measures of processing speed (d = 0.42), visual learning and memory (d = 0.92) and the composite (d = 0.80; Lewandowski et al. 2017).

Regarding pharmacological interventions, performance improvements in one or more cognitive domain were reported by several randomised pilot trials using small sample sizes. Three studies reported improvements in cognitive functioning of euthymic patients with BD using lurasidone (Yatham et al. 2017), intranasal insulin (McIntyre et al. 2012) and *Withania somnifera* (Chengappa et al. 2013) although findings from the *W. somnifera* study did not survive correction for multiple comparisons. Improvements in cognitive performance in bipolar depression was reported with erythropoietin (Miskowiak et al. 2014), mifepristone (Young et al. 2004; Watson et al. 2012) and creatine monohydrate (Toniolo et al. 2017). The creatine monohydrate study was exploratory, and the findings were not adjusted for multiple comparisons.

Finally, three studies evaluated the efficacy of neurostimulation in cognitive performance of patients with BD (Sciortino et al. 2021): two studies of repetitive Transcranial Magnetic Stimulation (rTMS) and one study of transcranial Direct Current Stimulation (tDCS). All three studies used a randomised double-blind sham-controlled design. One study (N = 43) failed to show pro-cognitive effects of active rTMS (20 sessions of left dorsolateral prefrontal cortex – DLPFC) over sham rTMS (Myczkowski et al. 2018). Conversely, the second rTMS study (N = 52) reported that patients who received 10 consecutive days of DLPFC rTMS had significant improvements in 2 out of 10 cognitive measures, namely, Wechsler Memory Scale-III Spatial Span (d = 0.80) and Category Fluency (d = 0.82) (Yang et al. 2019). However, these findings were not corrected for multiple comparisons. The tDCS study (N = 42) reported significant larger improvements in TMT-B (d = 0.98) and Rey-Osterrieth Complex Figure Test delayed recall measure (d = 0.35) in active versus sham tDCS (Bersani et al. 2017). Finally, a recently published (not included in the above review) pilot RCT (McIntyre et al. 2021) showed a significant improvement in verbal learning in active (N = 16) versus sham (N = 20) rTMS.

In sum, preliminary findings from a limited number of small studies suggest potential pro-cognitive effects of Cognitive Remediation, erythropoietin, intranasal insulin, lurasidone, mifepristone, rTMS and tDCS in BD.

## Discussion

In this review, we complied available evidence from systematic reviews and meta-analyses on various aspects of neurocognitive functioning in BD. Based on available data, the following conclusions can be drawn:

1. It is evident that many patients with BD exhibit significant cognitive impairment during all clinical states including euthymia, even after potential confounding factors such as demographic variables, the effects of medications and residual depressive symptoms are statistically controlled. Impairment is observed across multiple cognitive domains but appears to be more severe in verbal memory and executive functioning.
2. There is a considerable cognitive heterogeneity within BD and multiple cognitive subgroups including patients with intact or even above average cognitive abilities, those with selective or moderate impairment, and those with severe global impairment can be identified.
3. Residual depressive symptoms, poor clinical course and higher number of previous manic episodes may negatively impact cognitive performance in patients with BD. Data from 4 published studies suggest that history of childhood maltreatment can also negatively affect cognitive functioning in BD.
4. Evidence from a small number of studies suggests that treatment with lithium has minor negative impact on cognitive functioning in a subgroup of patients with BD who have suboptimal clinical response to that medication. Valproate seems to be associated with worse cognitive performance compared to other mood-stabilising medications while the impact of second-generation antipsychotics on cognition remains inconclusive.
5. 5-Clinical subtypes such as BD-I and BD-II, and history of psychotic features relate minimally to cognitive functioning, but cannot fully explain the observed cognitive heterogeneity in BD.
6. Diagnostic categories including BD, MDD and schizophrenia do not seem to correspond to distinguishable neurocognitive profiles, suggesting that neurocognitive deficit may be a symptom dimension which cuts across multiple diagnostic categories (Abramovitch et al. 2021). Nevertheless, severity of impairment appears to be most prominent in schizophrenia, less prominent in BD, and possibly least affected in MDD.
7. Current evidence suggest that in contrast to patients with schizophrenia, the majority of patients with BD have average or higher premorbid cognitive abilities. However, a subgroup of patients with BD has lower than average premorbid intellectual functioning which would suggest the impact of early neurodevelopmental factors in the development of BD in a subset of patients with BD.
8. Evidence from available prospective studies does not support the notion of progressive cognitive decline in BD beyond the first episode. On the contrary, some evidence exists to suggest patients may show some improvements in cognitive functioning with appropriate treatment following the first manic episode.
9. Cognitive functioning is a major predictor of occupational and day-to-day functioning in patients with BD and may have more impact than other clinical factors such as symptoms severity.
10. Preliminary findings from small studies have shown potential pro-cognitive effect of behavioural (Cognitive Remediation) as well as pharmacological (erythropoietin, intranasal insulin, lurasidone, and mifepristone) and neurostimulation (rTMS and tDCS) interventions. Large-scale RCTs for Cognitive Remediation (NCT04031560), lurasidone (NCT02731612), erythropoietin (NCT03315897), rTMS (NCT03217110), tDCS (NCT04296604) are under way to confirm the results of the pilot studies.

Findings from our review have implications for future directions: First, longitudinal studies of neurocognitive functioning in individuals who are at high risk of BD can provide a better understanding of the development and progression of neurocognitive impairment in BD, which would in turn lead to targeted early intervention strategies aiming at preventing or slowing the cognitive decline. Second, given the pivotal role cognitive abilities play in functional outcomes of patients with BD, future efficacy trials need to consider examining potential positive and negative impacts of interventions on cognitive performance. Finally, large-scale RCTs are needed to compare the pro-cognitive efficacy of various pharmacological and non-pharmacological interventions in different cognitive subgroups of patients at different stages of BD.
